# Presence of smooth muscle continuous with the rectal and vaginal walls in the deep perineal space prompts reconsideration of the deep transverse perineal muscle

**DOI:** 10.1038/s41598-025-09585-9

**Published:** 2025-07-03

**Authors:** Satoru Muro, Liu Chang, Suthasinee Tharnmanularp, Akimoto Nimura, Hiroshi Churei, Keiichi Akita

**Affiliations:** 1 Department of Clinical Anatomy, Graduate School of Medical and Dental Sciences, Institute of Science Tokyo, 1-5-45 Yushima, Bunkyo-ku, Tokyo, 113-8510 Japan; 2 Department of Masticatory Function and Health Science, Graduate School of Medical and Dental Science, Institute of Science Tokyo, Tokyo, Japan; 3 Princess Srisavangavadhana Faculty of Medicine, Chulabhorn Royal Academy, Bangkok, Thailand; 4 Department of Functional Joint Anatomy, Graduate School of Medical and Dental Sciences, Institute of Science Tokyo, Tokyo, Japan

**Keywords:** Deep transverse perineal muscle, Pelvic floor, Smooth muscle, Striated muscle, Musculoskeletal system, Muscle, Anatomy, Gastrointestinal system, Rectum

## Abstract

The deep transverse perineal muscle was considered a striated muscle within the deep perineal space; however, recent studies suggest that it consists of smooth muscle. We investigated the histological composition and spatial distribution of the deep perineal space and its anatomical relationships with surrounding structures to understand its role in the perineum. Perineal muscle shape and location from nine cadavers (five males; average age, 79 years) were macroscopically examined. Serial sections were histologically analyzed using Masson’s trichrome, Elastica Masson, and immunostaining of smooth muscle tissue, from which the structure was three-dimensionally reconstructed. An overall layered perineum structure was revealed in both sexes. A slender, band-like striated muscle was observed near the surface. Beneath this layer was a dense connective tissue membrane that provides separation. A triangular, plate-like shaped smooth muscle with transversely oriented fibers occupied the deep perineal space, which, unlike the striated muscle, was continuous with the rectal wall and was identified as the deep transverse perineal muscle. The smooth muscle was continuous with the longitudinal rectal muscle and, in females, with the vaginal muscular wall. Findings indicate that the deep transverse perineal muscle may be a distinct pelvic smooth muscle component.

## Introduction

The deep transverse perineal muscle (DTP) (musculus transversus perinei profundus) was likely first described by Luschka^1^ as a structure extending from the ischium to the perineal body, located dorsally to the greater vestibular gland^1^. Subsequent descriptions have been provided by Henle^2^ and Holl^3^. In the late 20th century and later, many anatomical textbooks described the DTP as a striated muscle in the deep perineal space, running transversely from the ischiopubic rami medially^4–7^.

Since the 1980 s, controversy regarding the existence of the DTP has remained unresolved, with some studies denying its existence^8–11^, others suggesting that it exists in males but not in females^12,13^, and others reporting that it is extremely rare and occurs at a very low frequency^14^. The cause of such discrepancies in interpretation is thought to stem not only from anatomical variation, sex differences, and developmental or age-related changes but also from the observer’s perception. The definition of the DTP has likely influenced the interpretation of the observed results. In general, the DTP is defined as a striated muscle located in the deep perineal space, with fibers running transversely; however, it is not necessarily imperative to adhere strictly to this definition. While clarifying the definition of the DTP is of secondary importance, the primary concern is identifying the structures present in the deep perineal space.

Recent reports have suggested that the DTP is composed of smooth muscles^15–20^, indicating the need to interpret the DTP’s nature without assuming that it is a striated muscle. Most anatomists agree on the existence of the perineal membrane (inferior urogenital diaphragm fascia), which separates the superficial and deep regions of the perineum, and the levator ani muscle, which separates the pelvic cavity from the perineum. Using these two structures (the perineal membrane and levator ani) as landmarks, we defined the region between the perineal membrane and the levator ani muscle as the deep perineal space. In this study, we chose an approach where we identified the main muscular structure occupying the deep perineal space and subsequently investigated its histological composition, spatial distribution, and relationship with surrounding structures. This study aimed to clarify the superficial-deep relationships and histological characteristics of perineal structures and reconsider the DTP’s nature.

## Methods

### Preparation of specimens

Nine cadavers (mean age at death, 79.0 years [range, 60–92]; five males and four females) were donated to our department in accordance with the Japanese Act on Body Donation for Medical and Dental Education (Act No. 56 of 1983). Prior to death, all donors voluntarily consented to the use of their remains as educational and study materials. Written informed consent was obtained from the donors after clearly explaining the purpose and methods of using the corpses. Following death, the informed consent form was explained to the bereaved families, who made no objections.

All cadavers were fixed via arterial perfusion with 8% formalin and preserved in 30% alcohol. Individuals with a history of pelvic organ surgery were excluded from the study.

### Macroscopic examination

Macroscopic examinations were performed on five specimens (three males and two females). The pelvic region was then excised en bloc. Muscles and connective tissues were sequentially dissected from the inferior side, and the three-dimensional structure and fiber direction were observed. First, the skin and subcutaneous tissues were removed, and the bone structure (pubic and ischial bones) and perineal muscles (bulbospongiosus, ischiocavernosus, superficial transverse perineal muscle [STP], and external anal sphincter) were identified. Next, the perineal muscles were gradually removed, and the perineal membrane was observed. Finally, the perineal membrane was removed to observe its structure more deeply.

### Immunohistological analysis

Histological analyses were performed on the remaining four specimens (two males and two females). A large area of tissue from the perineal triangle, including the urethra, vagina (in the case of females), and anterior wall of the rectum, was harvested en bloc. Histological examination of large tissue masses is called ‘wide-range serial sectioning’^21^. Tissue blocks were fixed by immersion in 10% formalin for 24 h and subsequently decalcified in Planck-Rychlo solution (AlCl_3_:6H_2_O 126.7 g/L, HCl 85 mL/L, HCOOH 50 mL/L) for 5 days. Following decalcification, neutralization was performed in 5% sodium sulfate for 12 h. Thereafter, the tissue blocks were dehydrated (70% ethanol, 80% ethanol, 90% ethanol, 100% ethanol, and xylene) for at least 24 h at each step. Subsequently, the blocks were embedded in paraffin for 5 days under negative pressure. The paraffin solution was changed thrice. Paraffin-embedded tissue blocks were serially sectioned into 5-µm-thick specimens at 1-mm intervals using a rotary microtome (RX-860; Yamato Kohki Industrial Co. Ltd., Saitama, Japan). The sections were oriented perpendicular to the perineal triangle (frontal section).

Histological sections were stained with Masson’s trichrome or Elastica Masson stain to identify muscular and connective tissues. Additionally, immunohistochemical staining of the sections was performed to confirm their distribution in the smooth muscle tissues. The slides were microwaved in 10 mM sodium citrate buffer (pH 6.0) for antigen retrieval. Endogenous peroxidase activity was inactivated by incubating tissues in methanol containing 0.3% H_2_O_2_ for 30 min. Nonspecific binding was blocked by incubation in phosphate-buffered saline (PBS) containing 0.05% Tween 20 and 2.5% goat serum at room temperature for 30 min. The sections were incubated overnight at room temperature with primary antibodies against smooth actin (ready-to-use actin, smooth muscle Ab-1, clone 1A4; Thermo Fisher Scientific) and skeletal myosin (1:200, NBP-1–89, 707, Myosin Heavy Chain 3 antibody, polyclonal; Novus Biologicals). The sections were washed and incubated for 30 min at room temperature with peroxidase-conjugated anti-mouse IgG (ready-to-use, MP-7452, ImmPRESSHRP goat anti-rabbit IgG polymer, Vector Laboratories, Burlingame, CA, USA) and anti-rabbit IgG reagents (ready-to-use, MP-7451, ImmPRESSHRP Goat Anti-Rabbit IgG Polymer, Vector Laboratories) as secondary antibodies. Immunocomplexes were detected using 3,3-diaminobenzidine (Fujifilm Wako Pure Chemical Corporation, Osaka, Japan) and counterstained with hematoxylin for 1 min. This immunological method is similar to our previous research methods for the pelvic floor and perineal region and is effective for detecting the distribution of smooth muscle tissues^22,23^.

### Three-dimensional reconstruction

Computer-assisted three-dimensional (3D) reconstruction was performed using serial histological sections obtained from two samples (one male and one female) from the histological examinations mentioned earlier. A total of 38 male and 37 female serial sections were used for 3D reconstruction, with an interval of 1 mm between each section in both cases. Stained histological sections were scanned as whole slides using a high-quality scanner (GT-X980; Seiko Epson Corp., Tokyo, Japan). The following structures were segmented on serial histological section images: bone (ischiopubic ramus), urethra, vagina (in females), bulb of the penis/bulb of the vestibule, Cowper’s gland/Bartholin gland, rectum (rectal circular and longitudinal muscles), STP, smooth muscle structure in the deep perineal space, external urethral sphincter, bulbospongiosus, ischiocavernosus, external anal sphincter, and levator ani. Segmentation was performed using the AI-assisted tool “Seg & Ref” (https://github.com/SatoruMuro/SAM2GUIfor3Drecon)^24^. The structure was then reconstructed from the segments using 3D Slicer (version 5.6.0; https://www.slicer.org/)^25^. After importing the mask images into 3D Slicer and setting the slice spacing in “Volume information,” the “threshold” function in “Segment editor” was used to extract the segmented area from the mask images. The 3D-reconstructed data were exported in STL format. The combination of wide-range serial sectioning and 3D reconstruction enables nondestructive 3D visualization of anatomical structures over a wide area^21,26^.

### Declaration of large language models in the writing process

During the preparation of this work, the authors used ChatGPT in order to improve the clarity and English language and grammar. After using this technology, the authors reviewed and edited the content as needed and take full responsibility for the content of the publication.

## Results

### Macroscopic examination

**Fig. 1:**
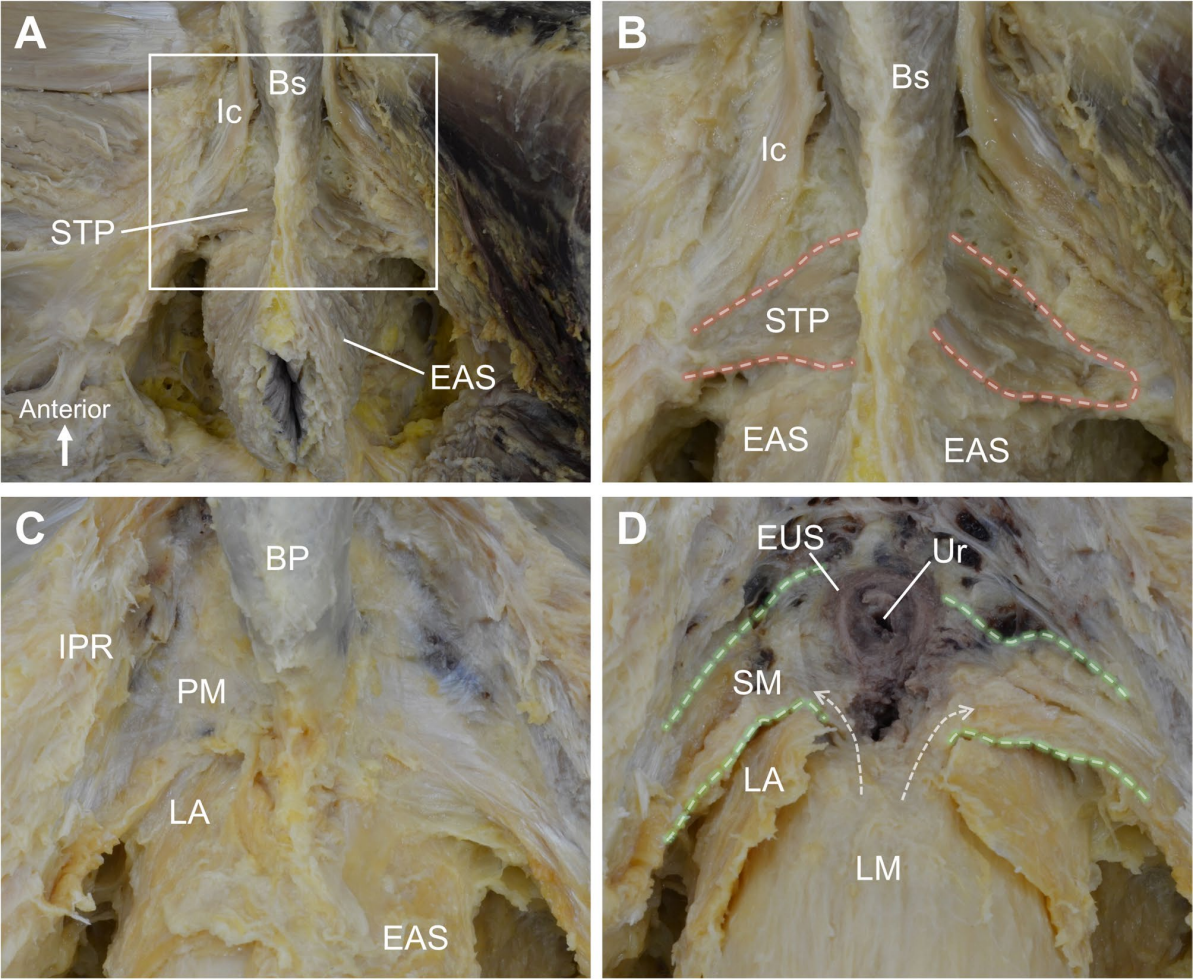
Macroscopic examination of the male perineum. (A) Removal of the skin, fat, and connective tissue reveals the perineal muscles. (B) A slender, band-like muscle extending from the midline to the IPR, identified as the STP. (C) Deeper dissection reveals the PM, a firm, dense connective tissue structure. (D) Removal of the PM exposes the structure, a triangular, plate-like shaped muscle with transverse fibers, continuous with the anterior rectal wall (dashed arrows). BP, bulb of penis; Bs, bulbospongiosus; EAS, external anal sphincter; Ic, ischiocavernosus; IPR, ischiopubic ramus; LA, levator ani; PM, perineal membrane; SM, smooth muscle; STP, superficial transverse perineal muscle; Ur, urethra.

In the male pelvis specimen, the removal of the skin, fat, and connective tissue of the perineum revealed the presence of perineal muscles (Fig. 1A). A slender, band-like muscle running from the midline to the ischiopubic ramus was observed, identified as the STP (Fig. 1B). Upon removal of the STP, a firm, dense connective tissue membrane was observed in the deeper layer and identified as the perineal membrane (Fig. 1C). After removing the perineal membrane, soft tissue extending laterally from around the urethra was observed. The tissue was triangular and plate-like in shape (Fig. 1D). The fibers ran in a transverse direction and were attached laterally to the ischiopubic ramus, situated inferior to the levator ani. No clear boundary was noted between these fibers and the longitudinal muscle of the anterior rectal wall, with the tissues appearing to be continuous (dashed arrows in Fig. 1D).

**Fig. 2:**
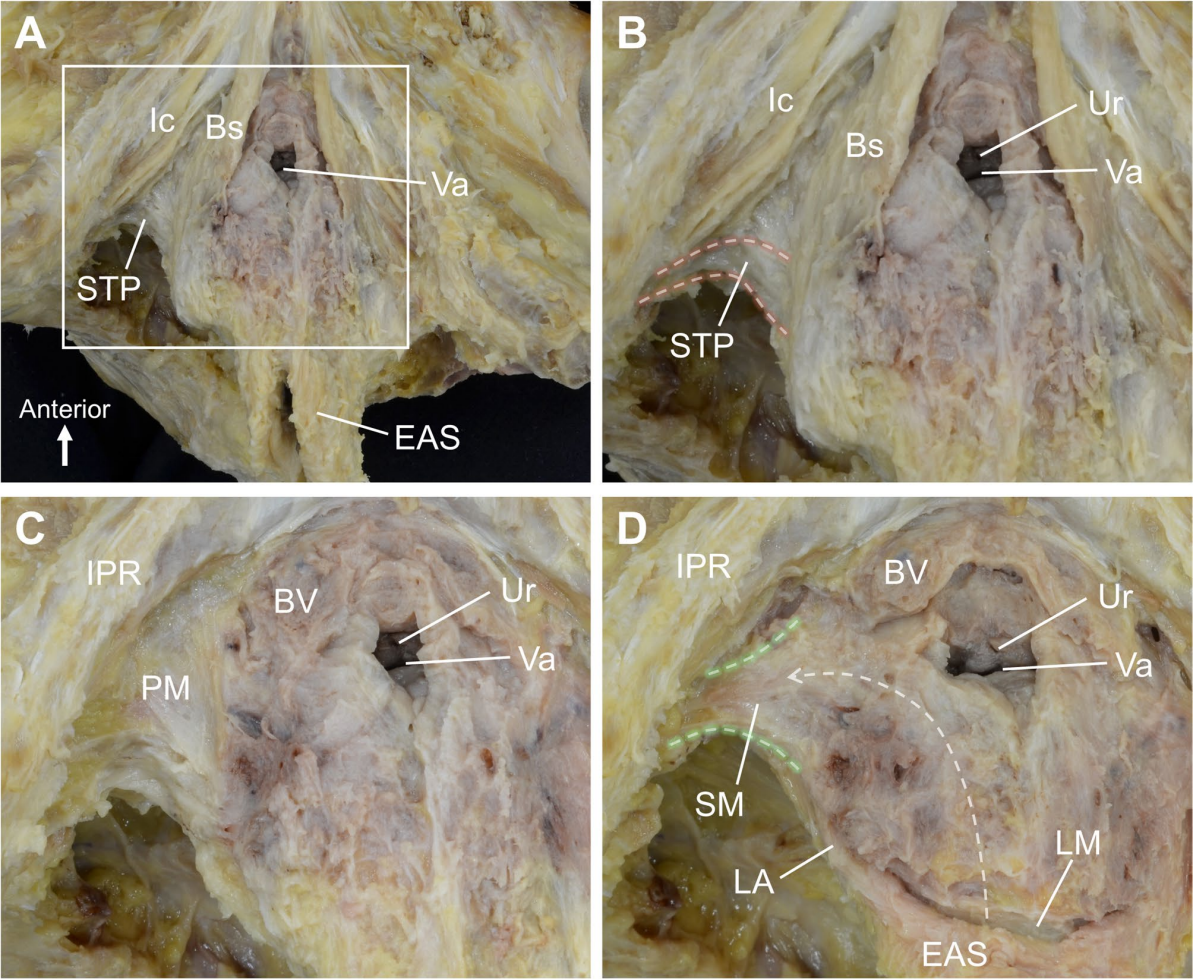
Macroscopic examination of the female perineum. (A) Dissection of the skin, fat, and connective tissue reveals the perineal muscles. (B) A slender, band-like muscle running transversely, identified as the STP, is located posterolateral to the vaginal opening. (C) Deeper dissection reveals the PM. (D) Removal of the PM exposes the structure, a triangular, plate-like shaped muscle with transverse fibers, continuous with the vaginal wall and anterior rectal wall (dashed arrow). BV, bulb of vestibule; Bs, bulbospongiosus; EAS, external anal sphincter; Ic, ischiocavernosus; IPR, ischiopubic ramus; LA, levator ani; PM, perineal membrane; SM, smooth muscle; STP, superficial transverse perineal muscle; Ur, urethra; Va, vagina.

In the female pelvis specimen, removal of the skin, fat, and connective tissue of the perineum revealed the presence of perineal muscles (Fig. 2A). A slender, band-like muscle running transversely was observed posterolateral to the vaginal opening, identified as the STP (Fig. 2B). The STP was attached laterally to the ischiopubic ramus and extended medially toward the midline, passing superior to the bulbospongiosus. Upon removal of the STP, a firm and dense connective tissue membrane structure was observed in the deeper layer, identified as the perineal membrane (Fig. 2C). After removing the perineal membrane, soft tissue extending laterally from around the vagina was observed. The tissue was triangular and plate-like in shape (Fig. 2D). The fibers ran in a transverse direction and were attached laterally to the ischiopubic ramus, situated inferior to the levator ani. No clear boundary was noted between these fibers, the vaginal wall, and the longitudinal muscle of the anterior rectal wall, with the tissues appearing continuous (dashed arrow in Fig. 2D).

### Immunohistological analysis

**Fig. 3:**
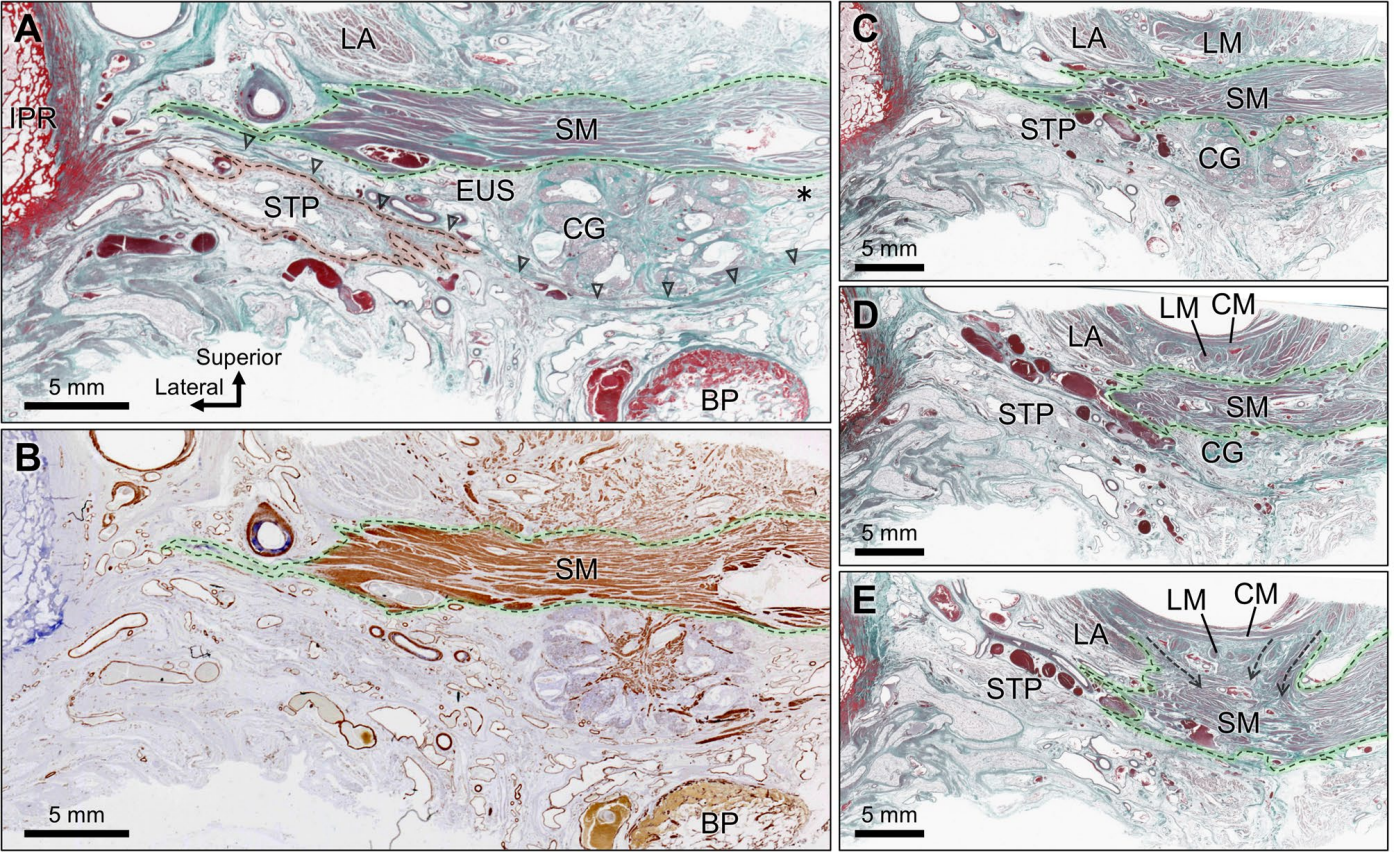
Histological sections of the male perineum (frontal section). (A) EVG Masson stain. The STP is observed running transversely, surrounded by superficial adipose tissue; the PM (arrowheads) and CG are seen superior to the STP. The plate-like structure is identified above the CG, with fibers running transversely. A small amount of striated muscle (asterisks) lateral to the CG was identified in later observations of serial sections as the posterolateral portion of the external urethral sphincter. (B) Immunohistochemical staining for smooth muscle confirms the presence of the smooth muscle structure in the deep perineal space. (C-E) Serial sections showing posterior progression: (C) is 3 mm posterior to (A), (D) is 2 mm posterior to (C), and (E) is 2 mm posterior to (D). These sections illustrate the continuity between the smooth muscle structure and longitudinal muscle fibers extending from the rectal wall (dashed arrows). BP, bulb of penis; Bs, bulbospongiosus; CG, Cowper’s gland; CM, circular muscle of rectum; EAS, external anal sphincter; Ic, ischiocavernosus; IPR, ischiopubic ramus; LA, levator ani; LM, longitudinal muscle of rectum; PM, perineal membrane; SM, smooth muscle; STP, superficial transverse perineal muscle; Ur, urethra.

In the histological specimen of the male perineum (frontal section), the STP was observed as a muscle running transversely, surrounded by the superficial adipose tissue of the perineum (Fig. 3A). The STP consists of striated muscle tissue that extends its fibers laterally toward the ischiopubic ramus. Superior to the STP, the perineal membrane is identified as a dense connective tissue membrane (arrowheads in Fig. 3A). The Cowper’s gland was located superior to the perineal membrane. Transversely running muscle tissue was observed above the CG. Lateral to the CG, a small amount of striated muscle tissue was observed (asterisk in Fig. 3A); however, due to its limited volume and because it did not constitute a primary component occupying the deep perineal space, it was not identified as the DTP. Observation of serial sections revealed that the striated muscle tissue represented the posterolateral portion of the external urethral sphincter (Fig. 5B). Immunohistochemical staining revealed the presence of smooth muscle in the deep perineal space (Fig. 3B). The levator ani was positioned superior to this structure (Fig. 3A). As the serial sections were traced posteriorly, the longitudinal and circular muscle layers of the rectal wall appeared medial to the levator ani (Fig. 3C-E). Fibers extending from the longitudinal muscle in the anteroinferior direction entered the smooth muscle structure in the deep perineal space (dashed arrows in Fig. 3E), indicating that the LM and this structure were histologically continuous.

**Fig. 4:**
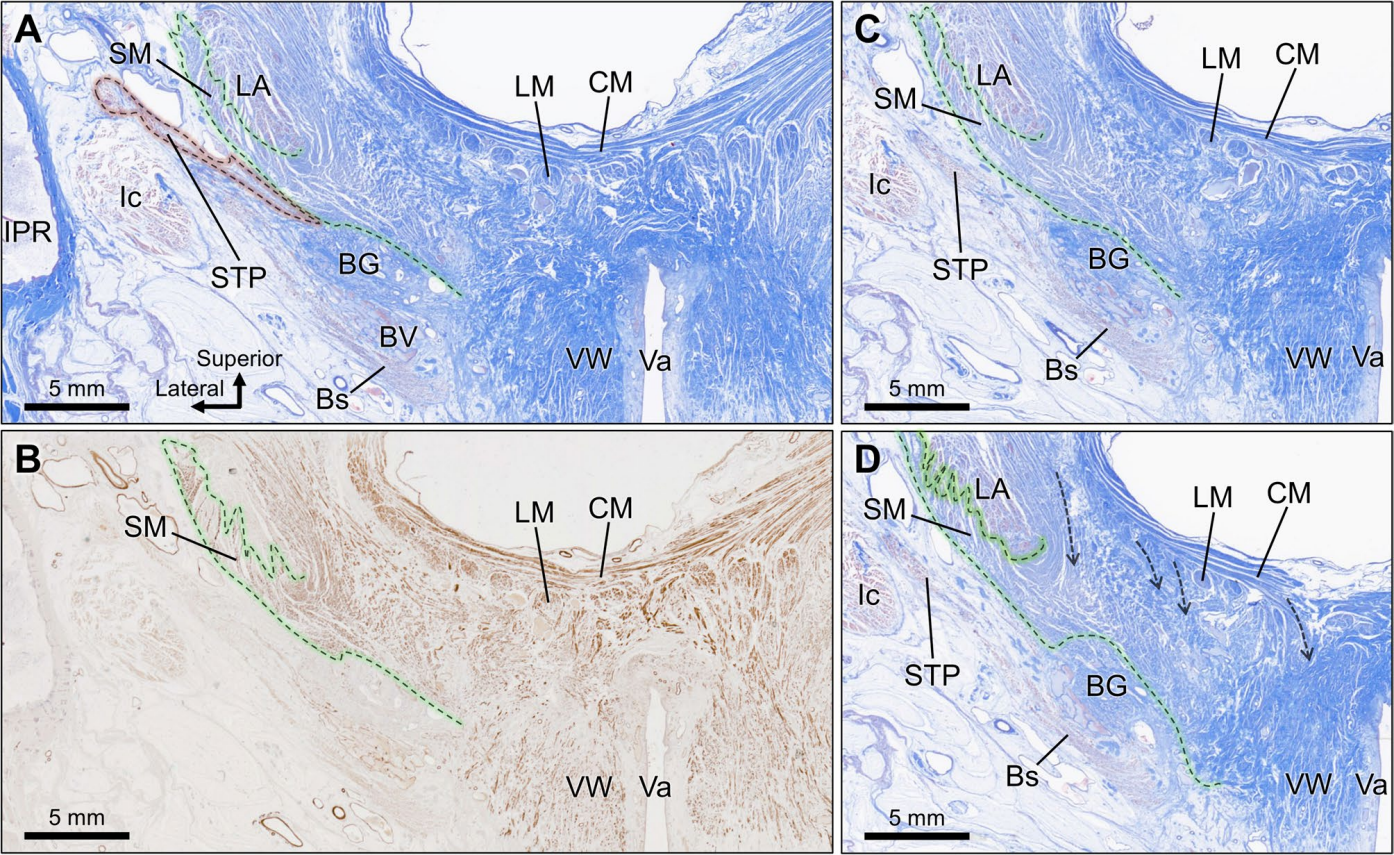
Histological sections of the female perineum (frontal section). (A) The STP runs transversely, positioned superior to the Bs; the plate-like structure is located above the STP. (B) Immunohistochemical staining reveals the presence of the smooth muscle structure in the deep perineal space. (C-D) Serial sections showing posterior progression: (C) is 1 mm posterior to (A), and (D) is 1 mm posterior to (C). These sections display continuity between the smooth muscle structure in the deep perineal space and LM fibers from the rectal wall (dashed arrows), as well as an indistinct boundary between the smooth muscle structure and VW. BG, Bartholin’s gland; Bs, bulbospongiosus; CG, Cowper’s gland; CM, circular muscle of rectum; EAS, external anal sphincter; Ic, ischiocavernosus; IPR, ischiopubic ramus; LA, levator ani; LM, longitudinal muscle of rectum; SM, smooth muscle; STP, superficial transverse perineal muscle; Ur, urethra; Va, vagina; VW, vaginal wall.

In the histological specimen of the female perineum (frontal section), the STP was observed to be transversely superior to the bulbospongiosus (Fig. 4A). The STP consists of striated muscle tissue that extends its fibers laterally toward the ischiopubic ramus. Laterally spreading muscle tissue was observed above the STP. Immunohistochemical staining revealed the presence of smooth muscle in the deep perineal space (Fig. 4B). The levator ani was positioned superior to this structure (Fig. 4A). Longitudinal and circular muscle layers of the rectal wall were observed medial to the levator ani. The relationships between the smooth muscle structure in the deep perineal space, rectal wall, and vaginal wall were examined by tracing serial sections posteriorly (Fig. 4C, D). Fibers extending anteroinferiorly from the longitudinal muscle entered the smooth muscle structure (dashed arrows in Fig. 4D), indicating continuity between the longitudinal muscle and this structure. Additionally, the boundary between the smooth muscle structure in the deep perineal space and the smooth muscle of the vaginal wall was indistinct, indicating that the two structures were histologically continuous.

**Fig. 5:**
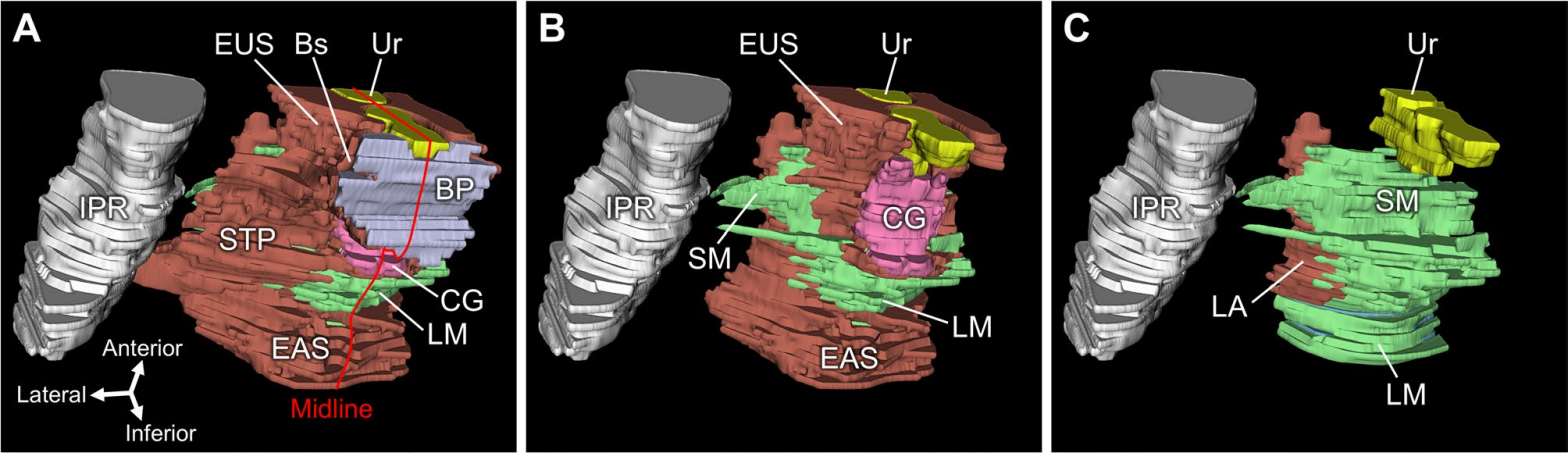
Three-dimensional reconstruction of the male perineum. (A) The STP extends from medial to lateral as a thin band in the superficial layer. (B) View after removal of the STP and BP. The smooth muscle structure in the deep perineal space, a triangular, plate-like structure in the deeper layer above the STP, is positioned anterior to the EAS and posterosuperior to the CG. (C) View after removal of the EUS, CG, and EAS, showing the continuity between the smooth muscle structure in the deep perineal space and LM. BP, bulb of penis; Bs, bulbospongiosus; CG, Cowper’s gland; EAS, external anal sphincter; EUS, external urethral sphincter; IPR, ischiopubic ramus; LA, levator ani; LM, longitudinal muscle of rectum; SM, smooth muscle; STP, superficial transverse perineal muscle; Ur, urethra.

### Three-dimensional reconstruction

The three-dimensional reconstruction allowed for the observation of the distribution of histological structures. In males, the STP extended medially to laterally as a thin triangular band in the superficial layer of the perineum (Fig. 5A). In the deeper layer (superior to the STP), the smooth muscle structure in the deep perineal space spreads as a triangular, plate-like structure (Fig. 5B). The external urethral sphincter and Cowper’s glands were located immediately below the smooth muscle structure. The smooth muscle structure was positioned anterior to the longitudinal muscle of the rectum, and both structures were continuous (Fig. 5C).

**Fig. 6:**
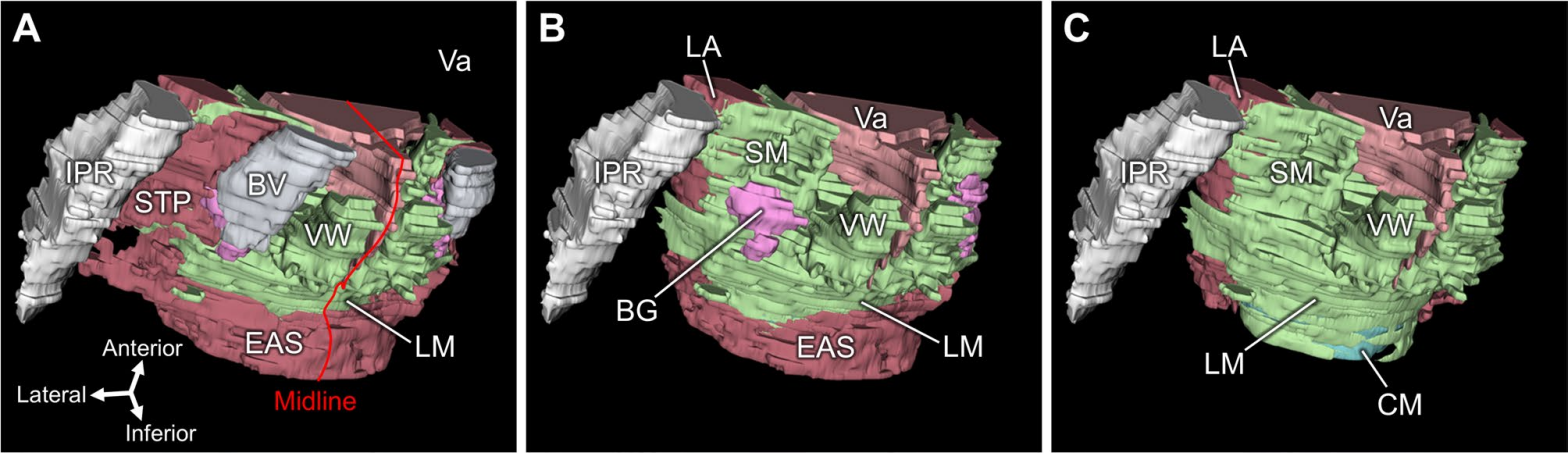
Three-dimensional reconstruction of the female perineum. (A) The STP extends laterally as a thin band in the superficial layer. (B) View after removal of the STP and BV, showing the smooth muscle structure in the deep perineal space, a plate-like structure positioned in the deeper layer above the STP and BG. (C) View after removal of the BG and EAS, showing the continuity between the smooth muscle structure in the deep perineal space, LM, and VW. BG, Bartholin’s gland; CG, Cowper’s gland; CM, circular muscle of rectum; EAS, external anal sphincter; EUS, external urethral sphincter; IPR, ischiopubic ramus; LA, levator ani; LM, longitudinal muscle of rectum; SM, smooth muscle; STP, superficial transverse perineal muscle; Ur, urethra; Va, vagina; VW, vaginal wall.

In females, the STP extended medially to laterally as a thin triangular band in the superficial layer of the perineum (Fig. 6A). The smooth muscle structure in the deep perineal space spread as a plate-like structure in the deeper layer (superior to the STP) (Fig. 6B). Bartholin’s gland was located immediately below the smooth muscle structure. The smooth muscle structure was situated lateral to the muscles of the vaginal wall and anterior to the longitudinal muscles of the rectum. These structures were continuous (Fig. 6C).

## Discussion

**Fig. 7:**
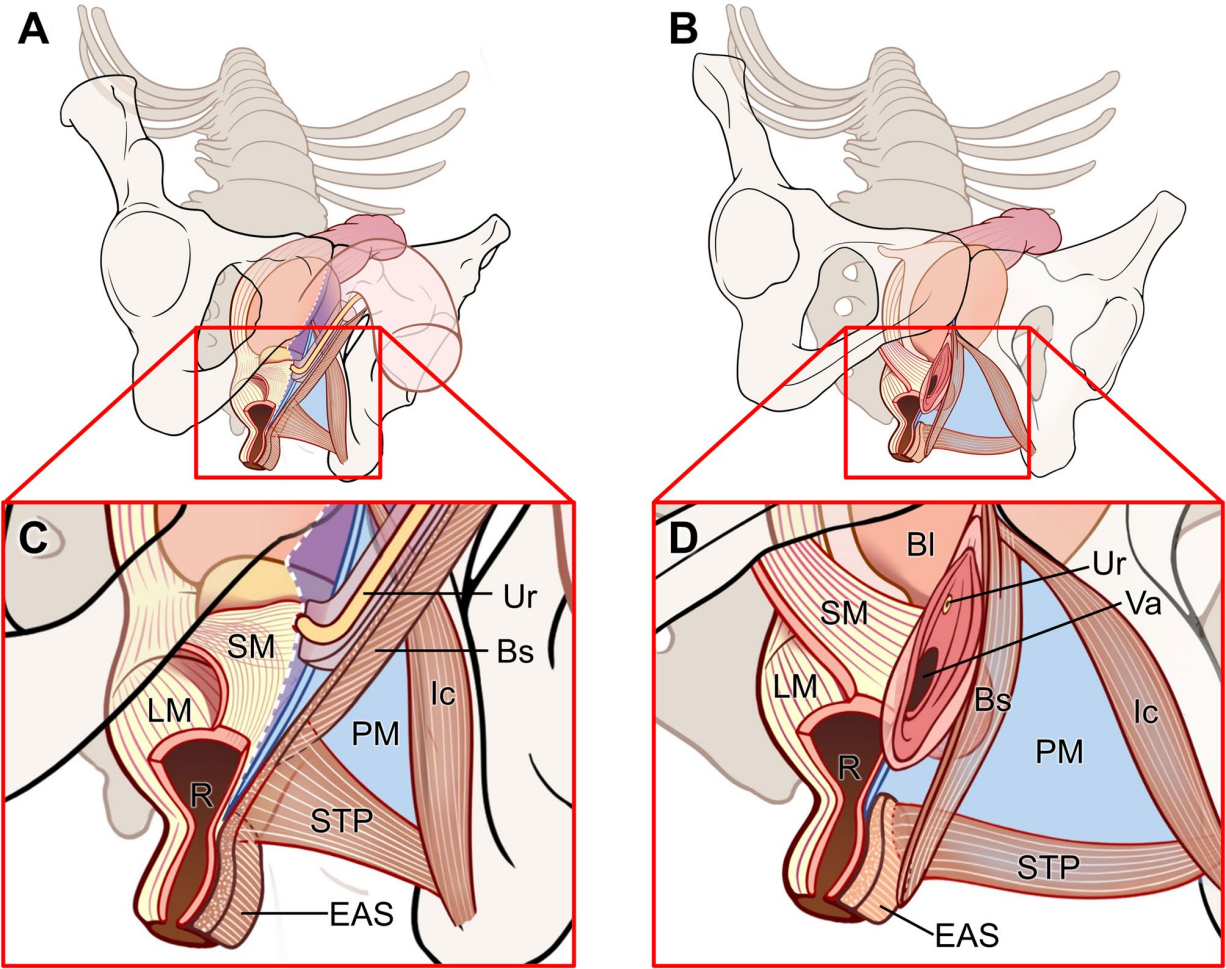
Schematic view of the pelvic region, seen from an oblique inferior angle. (A) Male pelvic structures. (B) Female pelvic structures. (C) Enlarged view of the male perineum, illustrating the arrangement from superficial to deep layers: the STP, followed by the PM, and then the smooth muscle structure in the deep perineal space, a triangular, plate-like shaped smooth muscle continuous with the rectal wall. (D) Enlarged view of the female perineum, showing the arrangement from superficial to deep layers: the STP, followed by the PM, and then the smooth muscle structure in the deep perineal space, a triangular, plate-like shaped smooth muscle continuous with both the rectal wall and the vaginal wall. Bl, bladder; Bs, bulbospongiosus; EAS, external anal sphincter; EUS, external urethral sphincter; Ic, ischiocavernosus; LM, longitudinal muscle of rectum; PM, perineal membrane; R, rectum; SM, smooth muscle; STP, superficial transverse perineal muscle; Ur, urethra; Va, vagina.

In both males and females, from the superficial to the deep regions of the perineum, there was a slender, band-like striated muscle, followed by a firm, dense connective tissue membrane, and then a triangular, plate-like shaped smooth muscle, with the levator ani muscle located cranially. The region previously thought to be occupied by striated muscle contained no plate-like structures of striated muscle; only smooth muscle was observed. The structure that primarily occupies the deep perineal space is a triangular, plate-like shaped smooth muscle with transversely oriented fibers. Therefore, this smooth muscle structure may correspond to the structure that has traditionally been referred to as the DTP. This structure is not an independent muscle but a smooth muscle structure continuous with the rectal wall and, in females, is also continuous with the vaginal wall (Fig. 7). These findings suggest a need to reconsider the concept of the so-called DTP in light of its histological characteristics and anatomical continuity with the rectal and vaginal walls.

As mentioned earlier, several studies have either denied or questioned the existence of the DTP^8–13^, while some reports have affirmed its existence^14,27^, but no consensus has yet been reached. The observations in these studies are accurate; however, because researchers have been searching for striated muscles in the deep perineal space, such as the DTP, differences in conclusions likely arose among observers^27–30^. As demonstrated in this study, in the adult human body, the structure that predominantly occupies the deep perineal space is composed of smooth muscles, and striated muscles rarely exist in the deep perineal space. Therefore, if the DTP is defined as the “striated muscle that predominantly occupies the deep perineal space,” the conclusion would be that the DTP does not exist. However, this does not imply that striated muscles do not exist in the deep perineal space. The external urethral sphincter is a striated horseshoe-shaped muscle located within the deep perineal space, with its posterior portion extending in the posterolateral direction^8,12,23,31,32^. The posterolateral part of the extending external urethral sphincter can be observed as transversely oriented striated muscle fibers in tissue sections (indicated by asterisks in Fig. 3A), which has led some previous studies to identify this region as the DTP^12^. However, because these striated muscles occupy only a small portion of the deep perineal space and are ultimately part of the external urethral sphincter or STP, it is not necessary to assign them a separate name (DTP). In contrast, this study did not focus on identifying the DTP as a striated muscle. Instead, we investigated the main structures constituting the deep perineal space and examined their positions, shapes, extents, and histological composition. Based on these criteria, the structure was identified as the DTP. We have previously reported the muscular sheet, which extended from the pelvic organs in the deep perineal space^15,16,33^, and in this study, we identified a triangular, plate-like structure composed of smooth muscle occupying the deep perineal space. Importantly, we confirmed that no striated muscle structure, which could be considered as the DTP, was observed. This finding suggests that the DTP may, in fact, consist of smooth muscle rather than skeletal muscle, prompting a re-evaluation of the conventional understanding of this region. Interpretation of this smooth muscle structure as corresponding to the so-called DTP is consistent with several recent reports^17–20^. Furthermore, an important finding of this study is that the smooth muscle structure is not an independent, isolated structure but a continuous extension of the longitudinal muscle of the rectum. We demonstrated tissue continuity, both anatomically and histologically.

The smooth muscle tissue in the deep perineal space, observed in this study, has sometimes been described under different names in previous reports. Some papers have referred to the smooth muscle tissue in the deep perineal space as the “perineal membrane”^10,34^, while other studies have called it the “lateral extension of the perineal body”^35^. Henle also noted that the deep perineal space (which he referred to as the urogenital diaphragm) contains a significant amount of smooth muscle tissue^2^. Similarly, Oelrich noted the presence of smooth muscle tissue in females^12^. However, there has been no established concept or terminology to describe smooth muscle structures in the deep perineal space; they have not been sufficiently recognized. The present findings may serve as a basis for reconsidering the anatomy of this region, particularly regarding the smooth muscle component that predominantly occupies the deep perineal space.

Given its position and shape, the smooth muscle structure—similar to the structure that has traditionally been referred to as the DTP—is likely a supportive structure of the urogenital triangle, functioning to prevent the widening of the urogenital hiatus. The continuity between the smooth muscle structure and the longitudinal muscle of the rectum also suggests that the smooth muscle structure is involved in defecatory function. Zhai suggested that it contributes to the formation of the anorectal angle and may play a role in fecal continence^20^. It is also suggested that the amount of smooth muscle tissue in the deep perineal space increases with age^13^, raising the possibility that this structure developed postnatally in humans, a species characterized by upright bipedal locomotion, to resist intra-abdominal pressure and gravitational forces. Our research group has previously shown that smooth muscle is widely distributed throughout the pelvic floor and perineum^15,16,36–45^. In the pelvic floor, smooth muscle extends outward from the pelvic organs and attaches to the supporting structures formed by striated muscle, creating a supportive framework in the form of a sheet. The fundamental components of the pelvic floor are striated and smooth muscles, which together form a cohesive support structure through a continuous connection: organs that extend the smooth muscle, smooth muscle attached to the striated muscle, and striated muscle attached to the bone. Therefore, this plate-like structure of smooth muscle is considered essential for connecting the striated muscles and supporting pelvic organs and is unique to the pelvic floor of humans.

This study has some limitations. First, the donated cadavers used in this study had an average age of over 70 years, indicating a relatively older population. Second, the parity status of the female cadavers was unknown, and thus the potential effects of parity-related anatomical changes in the deep perineal region could not be assessed. Third, the small number of specimens investigated may limit the generalizability of the findings, necessitating further research with a larger sample size. Fourth, this study did not investigate nerve supply to the muscles, which could provide further insight into their functional implications. Fifth, this study was purely anatomical; therefore, we could not provide quantitative measurements of the DTP movement. In the future, biomechanical studies may provide additional information.

## Conclusions

The structure primarily occupying the deep perineal space consists of smooth muscle with transversely oriented fibers, which may correspond to the structure traditionally referred to as the DTP. This smooth muscle structure is composed of tissue continuous with the longitudinal muscle of the rectum and, in females, with the vaginal muscular wall. Further, it appears to be a smooth muscle structure unique to the pelvic region and is involved in the support of the pelvic organs. These findings suggest a need to reconsider the anatomical concept of the so-called DTP, deepen our understanding of a novel anatomical structure involved in supporting pelvic organs, and are expected to serve as a foundation for future clinical applications and further research.

## Data Availability

Data supporting the findings of this study are available from the corresponding author upon request.
